# The spatiotemporal system dynamics of acquired resistance in an engineered microecology

**DOI:** 10.1038/s41598-017-16176-w

**Published:** 2017-11-22

**Authors:** Udaya Sree Datla, William H. Mather, Sheng Chen, Isaac W. Shoultz, Uwe C. Täuber, Caroline N. Jones, Nicholas C. Butzin

**Affiliations:** 1 Translational Biology, Medicine and Health, Virginia Polytechnic Institute and State University, Blacksburg, VA 24061 USA; 2 Center for Soft Matter and Biological Physics, Virginia Polytechnic Institute and State University, Blacksburg, VA 24061 USA; 3 Quantitative Biosciences, Inc., Solana Beach, CA 92075 USA; 4 Department of Physics, Virginia Polytechnic Institute and State University, Blacksburg, VA 24061 USA; 5 Department of Biological Sciences, Virginia Polytechnic Institute and State University, Blacksburg, VA 24061 USA; 6 Department of Biology and Microbiology, South Dakota State University, Brookings, SD 57007 USA

## Abstract

Great strides have been made in the understanding of complex networks; however, our understanding of natural microecologies is limited. Modelling of complex natural ecological systems has allowed for new findings, but these models typically ignore the constant evolution of species. Due to the complexity of natural systems, unanticipated interactions may lead to erroneous conclusions concerning the role of specific molecular components. To address this, we use a synthetic system to understand the spatiotemporal dynamics of growth and to study acquired resistance *in vivo*. Our system differs from earlier synthetic systems in that it focuses on the evolution of a microecology from a killer-prey relationship to coexistence using two different non-motile *Escherichia coli* strains. Using empirical data, we developed the first ecological model emphasising the concept of the constant evolution of species, where the survival of the prey species is dependent on location (distance from the killer) or the evolution of resistance. Our simple model, when expanded to complex microecological association studies under varied spatial and nutrient backgrounds may help to understand the complex relationships between multiple species in intricate natural ecological networks. This type of microecological study has become increasingly important, especially with the emergence of antibiotic-resistant pathogens.

## Introduction

Synthetic biology has emerged as a powerful tool employed in understanding the fundamental biological concepts and the prospective real world applications^1–3^. Using synthetic systems, researchers have been able to engineer better-defined cellular interactions and thus shed light on how these interactions lead to particular collective cell behaviours^4–16^. Especially, modelling of synthetic microecologies has allowed researchers to explore specific questions (e.g. biodiversity and coexistence of populations) using simplified models, the findings of which can be used to infer results about natural systems^17^. Most of the microbial association studies involve co-cultures and often fail to reflect the spatial relationship, which is important to study pattern formation^18–21^ and evolution. The first synthetic predator-prey ecosystem was constructed to study oscillatory population dynamics between predator and prey *E.coli* strains through the interaction of quorum sensing modules^22^. This system was then used to study the spatiotemporal modulation of biodiversity between two engineered *E. coli* populations^23^. Recently, a large step forward in the field was the analysis of the microbial evolution and growth arena (MEGA)-plate, where the spatiotemporal dynamics of microbial evolution of a single type of motile *Escherichia coli* was studied on an antibiotic background^24^.

In our current work, we developed a synthetic *E. coli* killer-prey ecosystem using two different non-motile *E. coli* strains to study the spatiotemporal dynamics of growth and acquired resistance *in vivo*. Our system is different from the canonical “predator-prey” system in that it is a unidirectional interaction where the killer inhibits the growth of the prey by constitutively secreting N-Acyl homoserine lactone (30C6HSL; AHL) (chemical-mediated), which at high concentrations results in the death of the prey. However, the killer does not directly benefit from the growth of the prey. This is due to the lack of competition between the killer and prey for nutrients since we used rich media in our experiments and non-motile *E. coli* strains which can utilise nutrients only in their fixed territory. Unlike previous systems, our design allowed us to focus on the evolution of a microecology from a killer-prey (susceptible prey) relationship to mutual coexistence of the killer and the resistant prey (that managed to evolve to be resistant to the effects of AHL secreted by the killer). To gain an overall picture of the killer-prey relationship and acquired resistance in the prey, we developed an ecological model emphasising the concept of the constant evolution of species. The killer-prey relationship and acquired resistance are explained by our mathematical model with simple rules relevant to natural ecological networks despite the simplicity.

## Results and Discussion

### Engineering of *E. coli* killer-prey synthetic system

**Figure 1:**
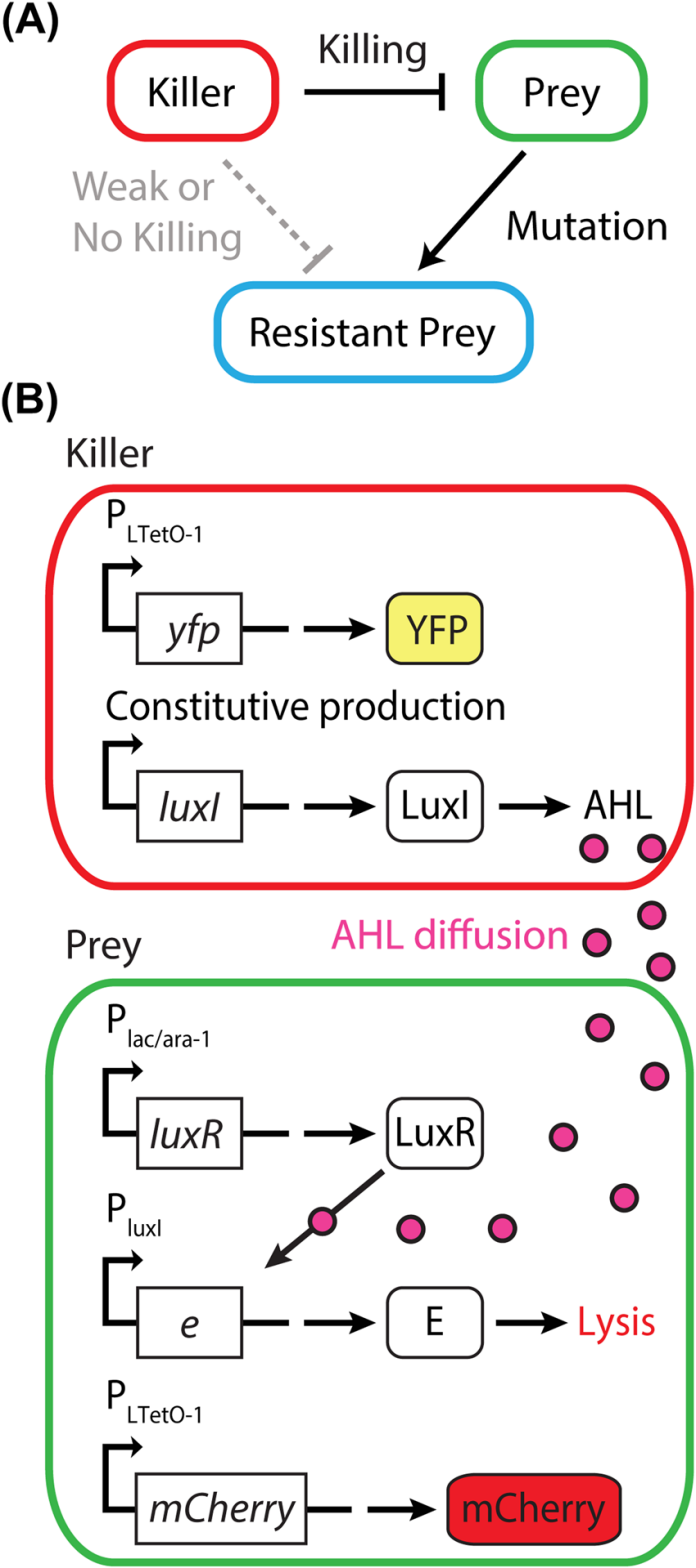
Schematics of *E. coli* killer-prey dynamics. (**A**) The conceptual figure shows the initial setup where the killer strain kills the prey strain; however, the prey can evolve to be resistant to the killer. (**B**) Two synthetic circuits, each on a different plasmid, allows for the killer and prey strain phenotypes. The killer constitutively produces LuxI, which functions to make AHL. AHL freely diffuses into the environment and enters the prey where it binds to the transcription factor LuxR (upregulated by the activation of P_lac/ara-1_ promoter in the presence of arabinose and IPTG) to form complexes. These complexes then activate the P_luxI_ promoter in prey to upregulate the expression of protein E to lyse the prey. The prey strain produces the repressor LacI (encoded in the genome), which is repressed by IPTG resulting in induction of P_lac/ara-1_ controlled genes. The killer strain does not contain the LacI repressor (not encoded in its genome), and thus *luxI* under the control of the P_lac/ara-1_ is constitutively produced. The system was set up in this manner so that the plasmid producing AHL (currently in the killer strain) could be inducible in other strains.

For our microecological studies, we engineered the killer and prey *E. coli* strains NB003 and DZ10, respectively. The killer strain secretes the quorum-sensing molecule N-Acyl homoserine lactone (30C6HSL; AHL) that freely diffuses out of the cell and into the environment. AHL can then enter prey and bind to the transcription factor LuxR (induced by arabinose and IPTG), and the formation of AHL-LuxR complexes then results in the production of a cell lysis protein E, which when produced kills the prey (Fig. 1). The killer strain was engineered to produce AHL constitutively, whereas the prey strain’s activity can be altered by the addition of arabinose and Isopropyl β-D-1-thiogalactopyranoside (IPTG), which then upregulate the downstream target genes necessary for quorum sensing. These strains were also engineered to produce fluorescent proteins (for tracking cells and for future studies); the killer constitutively produces YFP, a yellow fluorescent protein, while the prey produces a red fluorescent protein mCherry with the addition of doxycycline (see Methods section for details on strain construction). The killer strain lacks the ability to synthesise the amino acids leucine and isoleucine (engineered in these strains for future studies), which could allow nutrients released after prey lysis to feed killer, but we used rich media to avoid this mode of coupling in our current work.

### Spatiotemporal population dynamics of killer-prey in a nutrient-rich environment

Using our robustly designed microecological system, we then set out to study the spatiotemporal relationship between the killer and the prey. To achieve the killer-prey dynamics in nutrient-rich conditions, prey cells were smeared uniformly across LB (a rich media for *E. coli*) plates and then allowed to dry for approximately 10 to 15 min. This was then followed by patterning the killer cell population at the centre of the plate as a concentrated dot. To record time-lapse images, plates were scanned^25^ every hour.

**Figure 2:**
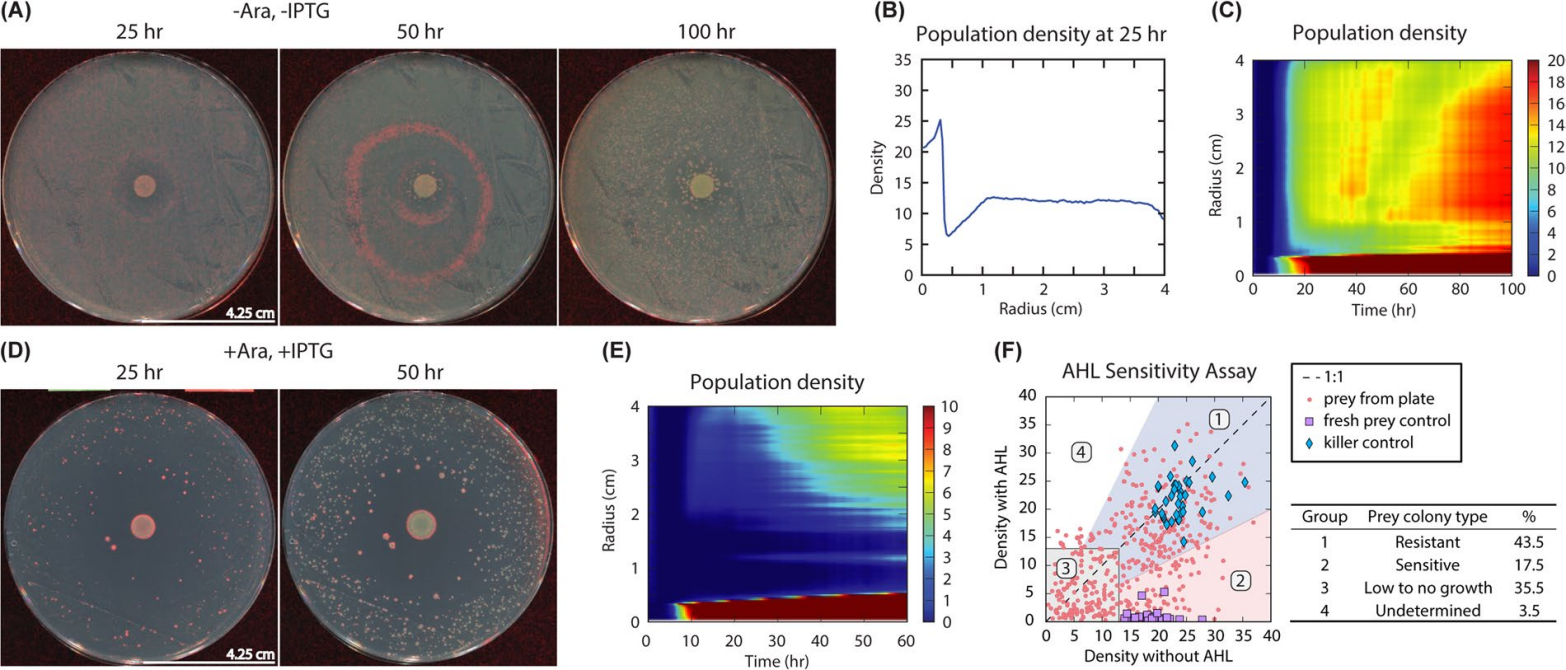
For all plating experiments, the prey culture was spread uniformly on the LB plate, left to dry for 10–15 min, and then the killer culture was dotted at the centre. (**A**) Snapshots from 25 hr, 50 hr, and 100 hr when cells were grown in the absence of both arabinose and IPTG (-Ara, -IPTG); see Video S4 for time-lapse experiment and Fig. S2 for detailed snapshots from the time-lapse experiment. A well-defined wave originated outside the killer colony, progressing through the prey lawn towards the periphery, which resulted in the formation of large prey colonies at ~100 hr. (**B**) The line plot shows the population distribution of *E. coli* grown on the plate in (A) (−Ara, −IPTG) at 25 hr. The region of less prey growth around the killer is represented as a dip in the plot. (**C**) The heat map shows the total population density of *E. coli* grown on the plate in (A) (−Ara, −IPTG) over a period of 100 hr. (**D**) Snapshots from Video S1 when cells were grown on plates with +Ara, +IPTG (inducers for LuxR which complexes with AHL in the prey). A *kill zone* is formed immediately surrounding the killer, and the prey population density increased with distance from the killer. (**E**) The heat map shows the total population density of *E. coli* grown on the plate in (**D**) (+Ara, +IPTG) over a period of 60 hr. (**F**) The AHL Sensitivity Assay consists of several steps (see Methods) where prey colonies (n = 434) were picked from various locations of the prey-killer +Ara, +IPTG plates (n = 3) and tested for viability on (−Ara, −IPTG) plates without AHL and then tested for resistance on the plates (+Ara, +IPTG) with AHL. Left: Scatter plot representing the frequency distribution of the four different prey groups (red circles) in the presence of the killer (+Ara, +IPTG): Group 1 consists of prey colonies (n = 189) resistant to AHL. Group 2 consists of colonies (n = 76) sensitive to AHL. Group 3 consists of colonies (n = 154) that had no apparent growth with and without AHL added. Group 4 consists of colonies (n = 15) that appear to have slightly more growth in the presence of AHL than without AHL. The killer control (blue diamonds) consists of fresh killer cells (n = 36; Fig. S4B), and the fresh prey control (purple squares) consists of prey not exposed to the killer or AHL (n = 36; Fig. S4A). Right: The percentages of the prey colonies from group 1–4. The dynamic patterns observed were repeatable; ≥4 biological replicates were tested. For all experiments with +Ara, +IPTG, 0.5% and 2 mM were used, respectively.

As expected, there was a substantial death of the prey by the killer in the presence of both arabinose and IPTG (+Ara, +IPTG; Fig. 2D). Interestingly, there was a spatial dispersion of the prey colonies all over the plate in relation to the killer cell population at the centre and the formation of a *kill zone* immediately surrounding the killer, beyond which the prey population gradually increased outward (Fig. 2D,E and Video S1; Table S1: Summary of time-lapse videos of prey and killer plates).

Unlike the previous condition, we observed a wave that originated outside the killer colony (at the centre of the plate), travelling towards the periphery when both arabinose and IPTG were lacking (−Ara, −IPTG). This suggests that the wave is of prey cells dying and we call this the “ring of death” due to its motion outward in a somewhat circular fashion. This wave of apparent death propagated through the prey after approximately 30 hr, and this wave was then followed by the emergence of round prey colonies both near and (to a lesser extent) far from the killer colony (Figs. 2A and S2; Video S4). The regions near the killer were sparse with respect to prey, but far regions supported prey growth. We supported this finding with quantitative image analysis of the time-lapse images of the total population density on the plate with time (Fig. 2B,C). We speculate that the round prey colonies that emerged following the *kill wave* were resistant to the effects of the AHL (at killer produced concentrations) and that their growth rate is proportional to their proximity to the killer.

We propose that the phenomena that were observed on the −Ara, −IPTG plates are a result of continuous secretion of the AHL by the killer at the centre and that the surviving prey colonies did so by acquiring resistance to this AHL-associated *kill wave*. This *kill wave* was observable on the −Ara, −IPTG plates and not on the +Ara, +IPTG plates perhaps due to the abundant number of prey cells in the absence of these inducers. The transcription factor LuxR is produced (Fig. 1) in the presence of arabinose and IPTG, which makes the prey cells more sensitive to the AHL; thus the density of the prey on +Ara, +IPTG plates is far less than the density on −Ara, −IPTG plates.

Controls were set up on the −Ara, −IPTG and +Ara, +IPTG plates to test if AHL produced by the killer resulted in prey death and if the prey was being killed by something other than AHL. On +Ara, +IPTG plates, when AHL was dotted at the centre instead of the killer (Fig. S1A; Video S2), similar effects (e.g. *kill zone*) were observed as that of the killer at the centre (Fig. 2D and Video S1). As expected, when prey was spread on +Ara, +IPTG plates without AHL or the killer strain, *kill zone* was not observed (Fig. S1B; Video S3). Meanwhile, on the −Ara, −IPTG plates with only prey, a wave arose at a random location on the plate propagating indefinably (Fig. S3; Video S5). Although we speculate the origin of this wave is due to local prey population density, there might be an underlying mechanism that we do not fully understand.

**Figure 3:**
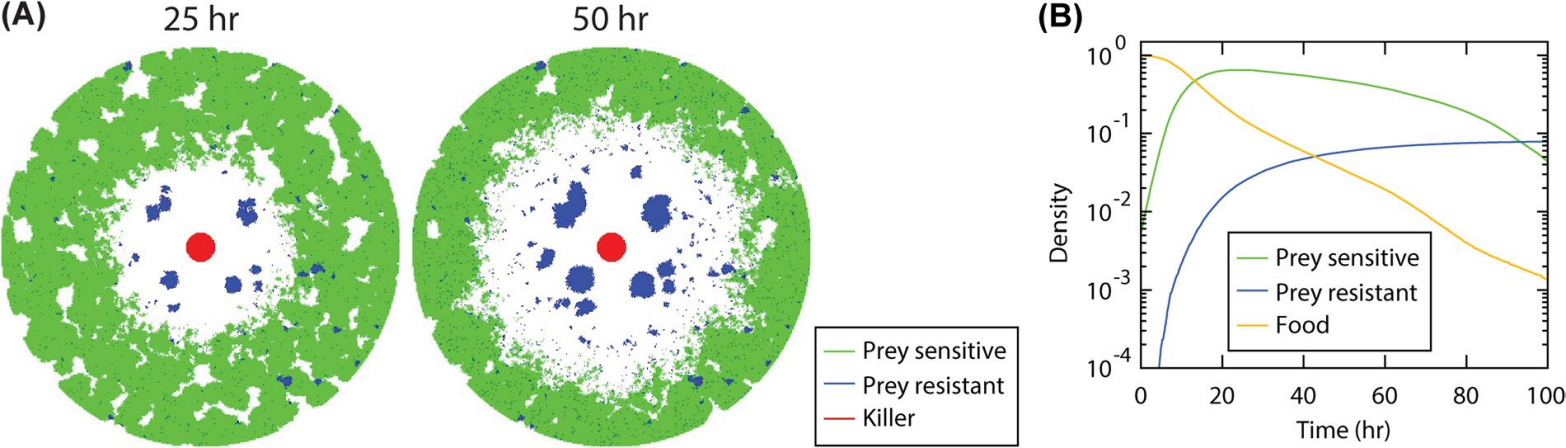
Monte Carlo simulations of the killer-prey system: The simulation was performed on a two-dimensional lattice with a circular absolute boundary with radius 200 lattice sites. On each lattice site, only at most one cell is allowed, i.e., one killer, one sensitive prey, or one resistant prey, while AHL and food particles can occupy the sites without any restriction. (**A**) Distribution of the particles when the system evolves for 25 and 50 hr, with the killers indicated in red, sensitive prey in green, and resistant prey in blue. AHL and food particles are not shown in the graphs. (**B**) Temporal evolution of the densities, obtained by dividing the total particle number with the number of total lattice sites, of the sensitive prey (green), resistant prey (blue), and food (yellow).

We know that the killer strain can kill susceptible prey, and we also know that the wave can be affected by the presence of the killer. However, the wave can be seen even in the absence of the killer strain; we speculate that this wave might then be related to a lysis response to the increased density of prey. We are not sure if the wave is AHL-based or somehow related to the leakiness of the P_luxI_ promoter, which changes in different growth phases or due to stresses. We do know that oxidative stress can induce the P_luxI_ promoter^26–28^ and it is reasonable to assume that oxidative stress increases with cell density. We do not know for sure what the wave is, but we identified several features of it. The wave can travel very fast (probably faster than AHL diffusion would explain), the wave is associated with susceptible prey that has grown as a thin but visible lawn, and the wave is sometimes followed up by the growth of resistant colonies (confirmed to be resistant prey by sensitivity assay, see Fig. 2F). We have only seen one wave total per experiment (patches of prey only undergo one wave per experiment in our studies), and based on the computational model (see Quantitative modelling section below; Fig. 3 and Video S17), it is thus reasonable to suggest that the wave is associated with cell death of susceptible prey followed by the growth of resistant prey.

To further probe into the robustness of this system, we set up four other *in vivo* experiments on −Ara, −IPTG plates (Table S1). (1) More than one killer was dotted on the plate (Video S12), and a wave originated outside each killer dot followed by the formation of several large prey colonies around each killer dot. (2) A line of killer cells was plated from end-to-end passing through the centre of the plate (Video S13), and a wave originated on either side of the killer line followed by the formation of several large prey colonies around the killer line. (3) All previous experiments used a prey to killer ratio of 1:1. We tested 1:2 and 2:1 ratios and we observed in these experiments a similar pattern to the 1:1 ratio (Videos S14 and S15, respectively). (4) We also tested plates with 13.5 cm diameter (Video S16), and the results were similar to the original (Fig. 2A and Video S4; previous experiments used plates with a diameter 8.5 cm).

### Acquired resistance in prey and exploring the resistant prey-killer population dynamics

We used an AHL Sensitivity Assay and sequenced selected colonies’ plasmid DNA, to test if they acquired resistance to the AHL through mutations. For the AHL Sensitivity Assay, isolated prey colonies were picked from various locations of the prey-killer +Ara, +IPTG plates 48 hr post-incubation and sequentially streaked on plates without AHL and then on plates with AHL (see Methods). The first plate tested the viability of the colony, while the second plate tested its response to a high concentration of AHL. As predicted, many of the colonies that survived on the original plate with the killer strain were resistant to AHL by the sensitivity assay (quantified in Fig. 2F). To demonstrate that the prey acquired resistance due to exposure to the killer strain, prey not exposed to the killer or AHL were tested using this assay, and those colonies were all (n = 36) sensitive to AHL (Fig. S4A). To demonstrate that the growth of *E. coli* cells without the death circuit (Fig. 1) is not hindered by the AHL concentration used in the AHL Sensitivity Assay, fresh killer cells from overnight cultures were tested, and the killer colonies grew similar to controls (n = 36; Fig. S4B).

We then analysed selected regions of the plasmid DNA from two resistant, five sensitive, and a control prey colony not exposed to the killer strain or AHL. As predicted, the later control colony had no apparent mutation in the E-protein gene. Neither did the five sensitive colonies. The two resistant colonies had an insertion of IS10 transposase (*tnpA*) gene^29^ in the E-protein gene (the two sequences matched over 750 bp of *tnpA*) confirming that these cells acquired resistance to killer and AHL through mutation. To show that the resistance was due to mutations, we chose to analyse only sequences of plasmids from two resistant colonies, and it seems likely to us that the other resistant colonies also contain mutations. However, their mutations may be located in a different part of the plasmid or in the chromosome, and the mechanisms causing those mutations could be different (e.g. point mutation, frameshift, etc.). The advantage of using a synthetic system like ours is that the entire system is located on a plasmid and we hypothesise that the majority of mutations resulting in resistance will occur in the plasmid sequence. Unlike natural systems that contain many factors with genes encoded throughout the genome, our synthetic system allows us to monitor mutations in a more tractable manner. Further studies of the constant evolution of microecologies using this or other synthetic systems may allow researchers to tune in on “evolutionary steps” in a more accountable manner.

Although the two resistant and five sensitive colonies’ DNA sequence and their response to the AHL Sensitivity Assay were as predicted, this information alone does not give us a measure of their long-term response to exposure to the killer. We thus asked, “Have the resistant colonies evolved to be completely resistant to the killer and have the sensitive colonies (even though they had no detectable change in the protein E gene sequence) evolved to respond to the killer in a different manner”? To answer these questions, we tested the two resistant prey colonies (Prey^R1^ and Prey^R2^; containing transposon insertions) and the two sensitive colonies (Prey^S1^ and Prey^S2^; previously exposed to the killer) against the killer (Table S1). As expected, the killer (dotted at the centre) had no apparent effect on either Prey^R1^ or Prey^R2^ growth (No *kill zone*, *kill wave*, or growth pattern formation due to growth defects) with or without arabinose and IPTG (Videos S6–S8). The results with neither of the sensitive prey colonies (Prey^S1^ and Prey^S2^) agreed with that of the wild-type prey in the presence of killer (Videos S9–S11), which is not too surprising. The sensitive colonies were exposed to some level of AHL produced by the killer on the original plates. The AHL Sensitivity Assay utilised a high concentration of AHL (Fig. 2F and Fig. S4), but the killer may produce AHL at a far lower level. Thus, the sensitive colonies may have a mutation elsewhere in the plasmid or in its genome that reduces their sensitivity to the concentration of AHL produced by the killer.

### Quantitative modelling of acquired resistance in prey population

To gain an overall picture of the killer-prey relationship and acquired resistance, we developed a mathematical model with very simple rules (see Methods) relevant to natural ecological networks despite its simplicity (Fig. 3; Video S17). This idealised model, representing the scheme of Fig. 1A was sufficient to reproduce the complex results seen in our experiments. An individual-based stochastic reaction-diffusion model on a two-dimensional circular lattice domain with the killer, sensitive prey, resistant prey, AHL and food species recapitulated both initial and final prey spatial patterns, including the dynamics of the emergence of growing resistant prey colonies in a manner similar to the experiment (Fig. 2D; Video S1). The Monte Carlo simulations incorporated the following reactions: A killer cell K may consume one food molecule (when available on its site) and produce an AHL molecule with probability μ = 0.1; with probability v = 0.02, either a sensitive prey cell P or mutated prey cell M consumes one food molecule and generates another P (or M) cell on one of its eight nearest neighbour sites if empty; a sensitive prey cell P may change to a mutated prey cell M with likelihood λ = 0.00001; if the mutation does not happen and there are AHL molecules on this site, this P would then be removed from the system. In addition, the food and AHL molecules randomly move to their neighbour sites during the simulation. Figure 3A shows the spatial cell distribution after the system has evolved for 25 and 50 hr, where a *kill zone* is formed around the killers and large resistant prey colonies are observed. Figure 3B displays the temporal evolution of the densities of the prey and food molecules (note the logarithmic scale). The number of food molecules decays monotonically since they are continuously consumed and not regenerated. Sensitive prey M increases in number during the initial 20 hrs of the simulation and decreases afterwards as a result of the lack of food and the increase of AHL molecules. The mutated prey population M maintains steady growth, since they are not subject to death processes, but with decreasing rate.

## Conclusion

Our mathematical modelling results aid us in the profound understanding of the evolutionary dynamics of the prey population in our engineered *E. coli* killer-prey system. It also helps us comprehend the correlation of biodiversity of the prey population in space in relation to a localised killer. This is important especially because it is not practical to investigate the same dynamics through wet-lab testing in such a short timespan owing to its complexity and large sample size. Besides, such lattice-based Monte Carlo simulations can be extended to study multiple prey-allies’ system dynamics in controlled environments. Such theoretical approaches possibly will provide insight into the population dynamics, emphasising on the spatiotemporal patterns of biodiversity in the context of natural ecosystems, and epidemiology^30^. They also shed light on the endangerment risk factor of a population subset (here, prey *E. coli*) in a natural ecosystem due to environmental contaminants/pollutants (AHL quorum-sensing molecule in this case).

The reduced complexity of synthetic ecologies allows for theoretical and experimental exploration of fundamental principles, which can be used concurrently to enhance our understanding of complex natural systems. The community-level properties exhibited by such engineered microbial consortia is not only important in answering the overarching questions of natural microecologies, but will also be substantial for advances in both medical and environmental research. Especially with increasing concerns about the emergence of antibiotic-resistant pathogens, our simple wet-lab experiments and underlining mathematical model may be used to unravel the spatiotemporal patterns of phenotypic and genotypic resistance acquired in these pathogens. This type of spatiotemporal dynamic experiments supported by mechanistic models may aid in ascertaining the strength of the microbial resistance when exposed to multiple drugs and different dosages. Furthermore, this approach can also be explored to study the crosstalk between the microbes of the microbiome in our gut. This, in turn, may be helpful in the development and characterization of a safe set of synthetic gut microbiome populations to treat patients suffering from certain gut related infections such as *C. difficile* ^31^. Our model could be further developed to uncover the intriguing details pertaining to spatiotemporal evolutionary dynamics in complex natural systems (using multiple synthetic prey-allies’ webs) under diverse environmental conditions such as temperature, availability of nutrients, oxygen level, degradation of secreted toxins, etc. Our system can be adapted to a range of organisms and challenges beyond acquired resistance.

## Methods

### Strains and Plasmids

*E. coli* DZ10 (prey) and NB003 (killer) strains were used for all *in vivo* experiments. Plasmid DNA sequences (in GenBank format as a single zip file) used in this work are provided in the Supporting Information. DZ10 was constructed from DH5alphaZ1 (purchased from Dr. Rolf Lutz)^32^ by introducing plasmids p14AmpNB16 and p31CmNB11. NB003 was constructed from NB001^33^ by introducing plasmids p14AmpNB20 and p31CmNB02. To construct p31CmNB11, a *mCherry* gene was PCR amplified from the mCherry Plasmid (gifts from Dr. Jeff Hasty, University of California, San Diego) using the primers B05m F (5′AAAGAGGAGAAAGGTACCATGGTGAGCAAGGGCGAG3′) and B05m R (5′CCTTTCGTTTTATGGTACCTCACTTGTACAGCTCGTCCATG3′), and this fragment was then cloned into p31Cm^34^ at a KpnI restriction site. To construct p31CmNB02, a *yfp* gene was PCR amplified from pNO-2CLAA (gifts from Dr. Jeff Hasty) using the primers B02y F (5′AAAGAGGAGAAAGGTACCATGTCTAAAGGTGAAGAATTATTCACTGG3′) and yfp N01 R (5′CCTTTCGTTTTATGGTACCTTATTTGTACAATTCATCCATACC3′), and this fragment was then cloned into p31Cm^34^ at a KpnI restriction site. To construct p14AmpNB16, the P_lac/ara-1_ *luxR* construct was purchased from ThermoFisher and cloned into the KpnI-PmeI restriction sites of p14AmpNB15. The p14AmpNB15 plasmid was constructed by cloning the P_luxI_ *Protein E* purchased from ThermoFisher into the ClaI-PstI restriction sites of p14Amp. Repeated attempts were made to clone the *Protein E* gene from the pEPop1 plasmid (gifts from Dr. Jeff Hasty), but the start codon for this gene was mutated in every colony sequenced (greater than ten colonies were sequenced). To minimise the potential mutation of the start codon, a synthetic construct was purchased from ThermoFisher that contained two adjacent start codons (ATGATG). To construct p14AmpNB20, the P_lac/ara-1_ *luxI* construct was purchased from ThermoFisher and cloned into the KpnI-ClaI restriction sites of p14Amp. The plasmid p14Amp was constructed by PCR amplification and cloning of a T1 terminator into KpnI-ClaI restriction sites of pZE14MCS (purchased from Dr. Rolf Lutz)^32^.

The plasmids p14AmpNB16, p14AmpNB20, p14AmpNB15, p14Amp, and pZE14MCS were maintained using ampicillin (Amp, 100 µg/ml), while p31CmNB11, p31CmNB02, p31Cm, and pEPop1 were maintained using chloramphenicol (Cm, 10 µg/ml). The cultures were grown in Lysogeny broth (LB), 0.2% glucose, and antibiotics.

The prey strain DZ10 produces two proteins (genes are on the genome), AraC and LacI, which are used to control the expression of genes through the P_lac/ara-1_ promoter. AraC is an activator in the presence of arabinose, while LacI is a repressor of this promoter. Isopropyl β-D-1-thiogalactopyranoside (IPTG) acts as a repressor of LacI, thus allowing for the activation of the P_lac/ara-1_ promoter. The addition of both arabinose and IPTG results in the expression of *luxR* from p14AmpNB16, the protein product (LuxR transcription factor) binds to free N-Acyl homoserine lactone (30C6HSL; AHL). The LuxR-AHL complex then induces expression of the protein E lysis gene (located on p14AmpNB16), which lyses the cell (Fig. 1).

The killer strain NB003 contains plasmid p14AmpNB20, which allows for the constitutive production of AHL. This was accomplished by using a strain lacking both *araC* and *lacI* genes in its genome, and placing the *luxI* gene (LuxI produces AHL enzymatically) under the control of the P_lac/ara-1_ promoter.

The prey and killer strains contain plasmids p31CmNB11 (mCherry) and p31CmNB02 (YFP) respectively, which allows for the production of fluorescent proteins as indicators. In the prey strain, mCherry is induced by the addition of doxycycline, while the killer constitutively expresses YFP, even though both are under the control of the P_LtetO-1_ promoter. The prey strain constitutively produces TetR (expressed from the genome), which is a repressor of this promoter. The addition of doxycycline represses TetR repression; thus doxycycline acts as an activator of the P_LtetO-1_ promoter. The killer strain genome does not contain *tetR*; thus it constitutively produces YFP. For all plate experiments, doxycycline (200 ng/ml) was added to the media.

### Killer-prey dynamics setup

Overnight cultures of prey (DZ10) and killer (NB003) were subcultured at 1:100 dilution and grown at 37 °C to an OD_600nm_ of about 0.3. The cultures were then subcultured at 1:3 dilution and grown at 37 °C to an OD_600nm_ of about 0.2. These cultures were then pelleted at 2500 × *g* for 10 min, the supernatant was discarded, and then the pellets were suspended to the original volume with LB and placed on ice. The prey culture was diluted to 1:64 and the killer culture was diluted to 1:2.8 with LB. In most cases, prey and killer cultures were plated at a final 1:1 ratio (based on OD_600nm_). To do this, 160 µl of the diluted prey culture was uniformly spread on LB plates containing 0.2% glucose, doxycycline (200 ng/ml), ampicillin (100 µg/ml), and chloramphenicol (10 µg/ml), and allowed to dry for 10–15 min. Then, 7 µl of the diluted killer culture was dotted at the centre of the plate (Fig. 2A,D, Fig. S2). The dilution and plating steps were repeated for all the biological replicates on both the plates with and without 0.5% arabinose and 2 mM IPTG (n = 4 and 6, respectively). The plates were placed in a commercial flatbed Epson Perfection V37/V370 photo scanner at 37 °C and adjusted to capture time-lapse images at regular intervals of 1 hr.

Several controls were used to confirm our findings as described above. The prey without killer or AHL was spread across the plates with and without 0.5% arabinose and 2 mM IPTG (n = 3 and 5, respectively; Figs. S1B and S3). To demonstrate that AHL caused the death of the prey, a control with AHL (7 µl of 160 µg/ml) dotted at the centre instead of the killer was tested on plates with 0.5% arabinose and 2 mM IPTG (n = 4; Fig. S1A). All n values correspond to different biological replicates.

### Construction and analysis of time-lapse videos of killer-prey dynamic relationship

The time-lapse images captured via the scanner were analysed using in-house scripts. This method was altered and adapted from a previous group that used a flatbed scanner to quantify bacterial growth dynamics^25,35^. Images from scanners were acquired at a resolution of 600 dpi. Data was taken with a frequency of one image frame per hr. Frames were aligned at a single pixel resolution using a standard image registration algorithm based on fast Fourier transforms.

False-coloured images in videos S1 and S4 were highlighted by adding to the original images a red overlay image that was an estimate of the rate of change in the image. To compute the overlay image, we first computed the difference between each image and its prior image (for red, green, and blue channels separately), then we applied a spatial median filter with a 5 × 5 pixel window (for red, green, and blue channels separately), and we then computed a running median of the most recent four such different images (spanning in total 5 hr). This produced a smooth estimate of the rate of change of brightness for red, green, and blue channels. To compute the final overlay image, we summed the absolute value of this filtered difference image across red, green, and blue channels, and we then multiplied the resulting overlay image by a scaling constant before adding it to the raw acquired image. The sum of the raw acquired image and the overlay image is displayed in applicable videos.

### Construction of heat maps

Radial distribution analysis of time-course experiments were performed as follows. The location of the plate centre (corresponding to the centre of the killer colony, or alternatively where AHL was spotted) was first determined manually in Fiji^36^. The mean value of the green pixel channel was determined for regions corresponding to concentric two-pixel thick circles centred on the plate centre. Finally, for each radius, we subtracted from the radial intensity a background value, with background determined by the median radial intensity over the first five hr of the experiment (before noticeable growth was seen). Figure 2C,E are heat maps of killer-prey dynamics on −Ara, −IPTG and +Ara, +IPTG plates respectively after background values were subtracted. All other heat maps were constructed in a similar manner.

### AHL Sensitivity Assay and sequencing

All LB plates used for the Sensitivity Assay contained 0.2% glucose, doxycycline (200 ng/ml), ampicillin (100 µg/ml), and chloramphenicol (10 µg/ml). Prey colonies (n = 434) were picked from various locations of three different biological replicates of prey-killer plates (containing both arabinose and IPTG) after 48 hr of incubation. Individual colonies were streaked on the LB plates without arabinose, IPTG, and AHL, and then successively streaked on the LB plates with 0.5% arabinose, 2 mM IPTG, and 160 µl AHL (160 µg/ml). Before streaking colonies, the AHL was spread on the plates and allowed to dry for 10–15 min. Plates were incubated at 37 °C for 48 hr. As controls for AHL-sensitivity and AHL-resistance, this assay was also performed with both fresh prey and fresh killer, respectively (n = 36 for each) (Fig. S4). The interpretation of the results was done by comparing the intensity of growth on the plates with and without AHL, using our in-house script.

From select prey colonies, plasmid DNA was isolated and sequenced at the Biocomplexity Institute of Virginia Tech using primers USD F2 (5′TGATCTTCAGCATCTTTTACTTTCACC3′) and USD R1 (5′ACCGCCTTTGAGTGAGCTGATA3′).

### AHL assay image analysis

In order to quantify the prey colonies from the prey-killer plates into groups based on the AHL Sensitivity Assay, we analysed the growth on −AHL and +AHL plates (Fig. 2F). Images for our AHL assay were automatically analysed using a custom Fiji macro^36^. This macro first subtracted a constant value from each image (with negative values clipped to zero), and the macro then multiplied the image by a constant value. This procedure produced an image that was bright primarily where developed colonies existed. The manually-guided analysis then calculated the mean grey value within each of the boxes for the 6 × 6 square grid on the petri dish. Four different groups were identified based on the analysis. Group 1 consists of prey colonies (n = 189) that are resistant to AHL. Group 2 consists of colonies (n = 76) that are sensitive to AHL. Group 3 consists of colonies (n = 154) that had no apparent growth with and without AHL added. Group 4 consists of colonies (n = 15) that appear to have slightly more growth in the presence of AHL than without (Fig. 2F).

### Software for image analysis

Image analysis was done in Python using custom scripts and standard routines in the SciPy library.

### Modelling and simulation algorithm

We used a spatially extended stochastic simulation on a two-dimensional square lattice with approximately π*200^2^ sites, subject to circular absolute boundary conditions at a radius 200 lattice sites from the centre of the lattice. We impose occupation restrictions at each lattice site: at most one of the killer (K), prey (P), or mutated prey (M) can exist at a site. Food (F) and AHL (A) molecules may exist in any abundance at each site. The individual particles in the system undergo stochastic reactions as implemented using efficient Monte Carlo techniques developed for the study of lattice-based systems^37–39^. The reactions used for the algorithm are as follows with specific values μ = 0.1, v = 0.02, and λ = 0.00001 used for the purposes of simulation in Fig. 3 of the main text:

production of AHL by killer: K + F → K + A with probability μ,

reproduction of prey: P + F → P + P with probability v,

reproduction of mutated prey: M + F → M + M with probability v,

mutation of prey: P → M with probability λ,

killing of prey by AHL: P + A → A with probability 1 − λ.

Details concerning how these reactions are used are included below.

The simulation algorithm runs for a predetermined number of Monte Carlo Steps (MCS). One MCS corresponds to when on average all K, P, and M particles have participated in the above reactions once. That is, if N cells (K, P, or M) exist at a current point in time, then the simulation is advanced a duration 1.0/N when one of K, P, or M is selected as tested to react according to the details of the algorithm. This scales time-based on population number.

The detailed Monte Carlo algorithm for the stochastic reactions is as follows:

1. Select a lattice occupant (K, P, or M, do not include food or AHL) at random and generate a random number r uniformly distributed in the range [0, 1].
  - If it is a killer cell K with food molecules available on this site and r < μ, decrease the food molecule number of this site by 1 and then increase the AHL number by 1.
  - If it is a mutated prey cell M with food molecules available on this site and r < v, it consumes one food molecule and generates another M cell on one of its eight nearest neighbour sites if empty.
  - If it is a normal prey cell P, there is a probability λ that this P cell changes to an M cell. If the mutation does not happen and there are AHL molecules on this site, this P would then be killed and the state of the site becomes empty (with food and AHL molecules still on the site).
  - If neither the mutation nor the death occurs, this P cell may reproduce another P cell on one of its empty neighbour sites with a probability v by consuming a food molecule.
2. If there are food or AHL molecules on this site after the above reactions, each molecule has a probability D to diffuse to the neighbour sites. In the simulation, the value of D is fixed to 1.0. In this case, all the molecules at this site would diffuse to neighbour sites if no other reactions occur.
3. Return to step (1) until a certain number of MCS have been completed.

## Electronic supplementary material

Video S1

Video S2

Video S3

Video S4

Video S5

Video S6

Video S7

Video S8

Video S9

Video S10

Video S11

Video S12

Video S13

Video S14

Video S15

Video S16

Video S17

Supplementary Information
