# Supplementary material for: The spatiotemporal system dynamics of acquired resistance in an engineered microecology
Source: Sci Rep. 2017 Nov 22;7:16071. doi: 10.1038/s41598-017-16176-w (PMC5700104; doi:10.1038/s41598-017-16176-w)
Supplement: Supplementary file 18 — Supplementary Information [file 41598_2017_16176_MOESM18_ESM.pdf]

The spatiotemporal system dynamics of acquired resistance in an engineered microecology

Udaya Sree Datla[1,2], William H. Mather[3], Sheng Chen[2,4], Isaac W. Shoultz[2,4], Uwe C. Täuber[2,4], Caroline N. Jones[5] and Nicholas C. Butzin*[6].

[1]Graduate program in Translational Biology, Medicine and Health, Virginia Polytechnic Institute and State University, Blacksburg, VA, 24061, USA.
[2]Center for Soft Matter and Biological Physics, Virginia Polytechnic Institute and State University, Blacksburg, VA, 24061, USA.
[3]Quantitative Biosciences, Inc., Solana Beach, CA, 92075, USA.
[4]Department of Physics, Virginia Polytechnic Institute and State University, Blacksburg, VA, 24061, USA.
[5]Department of Biological Sciences, Virginia Polytechnic Institute and State University, Blacksburg, VA, 24061, USA.
[6]Department of Biology and Microbiology, South Dakota State University, Brookings, SD, 57007, USA.

*Corresponding author:
Nicholas C. Butzin
Department of Biology and Microbiology
South Dakota State University
1224 Medary Ave
Brookings, SD 57007
Email: nicholas.butzin@gmail.com
Phone: 605-688-4078
Fax: 605-688-6677
Section: Research article
Keywords: synthetic biology, killer, prey, microbial ecology, evolution

Table S1. Summary of time-lapse videos of prey and killer plates. Unless stated otherwise, the prey was spread across the plate, set to dry for 10-15 min, and then the killer or AHL was dotted at the centre of the plate. Following the AHL Sensitivity Assay (Fig. 2F), two colonies that were sensitive ($Prey^{S1}$ and $Prey^{S2}$) and two colonies that were resistant to a spread of AHL (160 µg/ml) ($Prey^{R1}$ and $Prey^{R2}$; both contain a transposon insertion in the E-protein gene) were plated again with the killer strain. In many experiments, we observed waves that may be due to prey dying. Waves generally originated outside the killer colony and travelled towards the periphery. Waves were only observed (after approximately 30 hr) after the prey grew into a lawn.

Video	Related Fig.	Prey: killer ratio*	Spread	Centre dot	Ara/IPTG	Wave formation
S1	2D-E	1:1	Prey	Killer	+/+	No
S2	S1A	NA	Prey	AHL	+/+	No
S3	S1B	NA	Prey	None	+/+	No
S4	2A-C, S2	1:1	Prey	Killer	-/-	Yes, originated outside the killer colony
S5	S3	NA	Prey	None	-/-	Yes, arose at seemingly random locations
S6		1:1	$Prey^{R1}$	Killer	-/-	No
S7		1:1	$Prey^{R2}$	Killer	-/-	No
S8		1:1	$prey^{R2}$	Killer	+/+	No
S9		1:1	$Prey^{S1}$	Killer	-/-	Yes
S10		1:1	$Prey^{S2}$	Killer	-/-	No
S11		1:1	$Prey^{S2}$	Killer	+/+	No
S12		1:1	Prey	2 Killers**	-/-	Yes, originated outside each killer dot
S13		1:5	Prey	Killer as a line along the diameter	-/-	Yes, originated on either side of the killer line
S14		1:2	Prey	Killer	-/-	Yes
S15		2:1	Prey	Killer	-/-	Yes
S16***		1:1	Prey	Killer	-/-	Yes
S17		1:1	Virtual Prey	Virtual Killer	+/+	NA (Lattice-Based Model)

* Prey: killer ratio was based on $OD_{600\ nm}$
** The two killers were dotted about 4.2 cm apart on the diameter and equally spaced from the centre.
*** All experiments were done on circular plates with 8.5 cm diameter, except in video S16, where the experiment was done on a plate with 13.5 cm diameter. The same number of killer and prey were used on all plates at 1:1 ratio.
NA: not applicable.

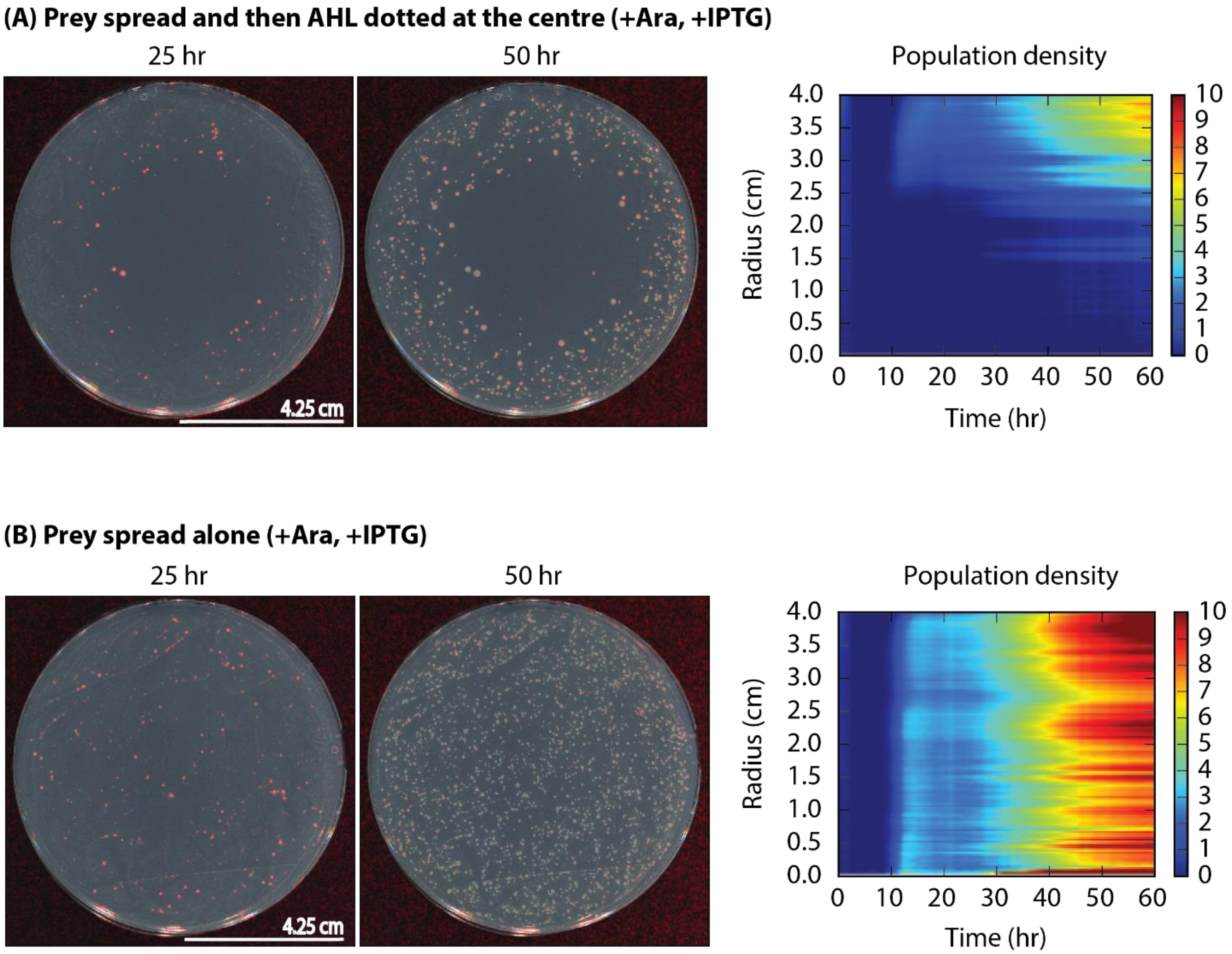

Fig. S1. (A) Left: Snapshots from Video S2 when AHL was dotted at the centre instead of the killer on the spread prey (+Ara, +IPTG). Right: Heat map showing the total population density of *E. coli* grown on the same plate over a period of 60 hr. **(B)** Left: Snapshots from Video S3 when the prey grew into a lawn (+Ara, +IPTG) without AHL or the killer. Right: Heat map showing the total population density of *E. coli* grown on the same plate over a period of 60 hr. See Methods for the experimental setup. The dynamic patterns observed were repeatable; $\geq$ 3 biological replicates were tested.

Prey spread and then killer dotted at the centre (-Ara, -IPTG)

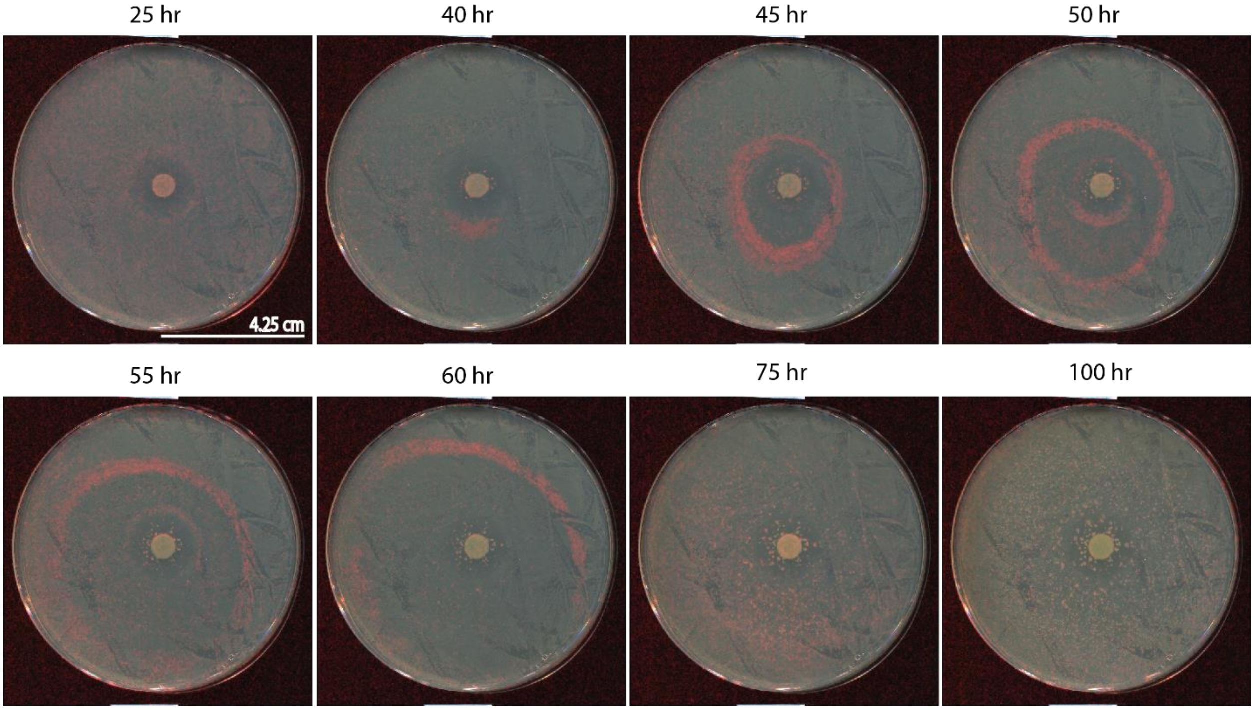

Fig. S2. Detailed snapshots from Video S4 as described in Fig. 2A and Methods.

Prey spread alone (-Ara, -IPTG)

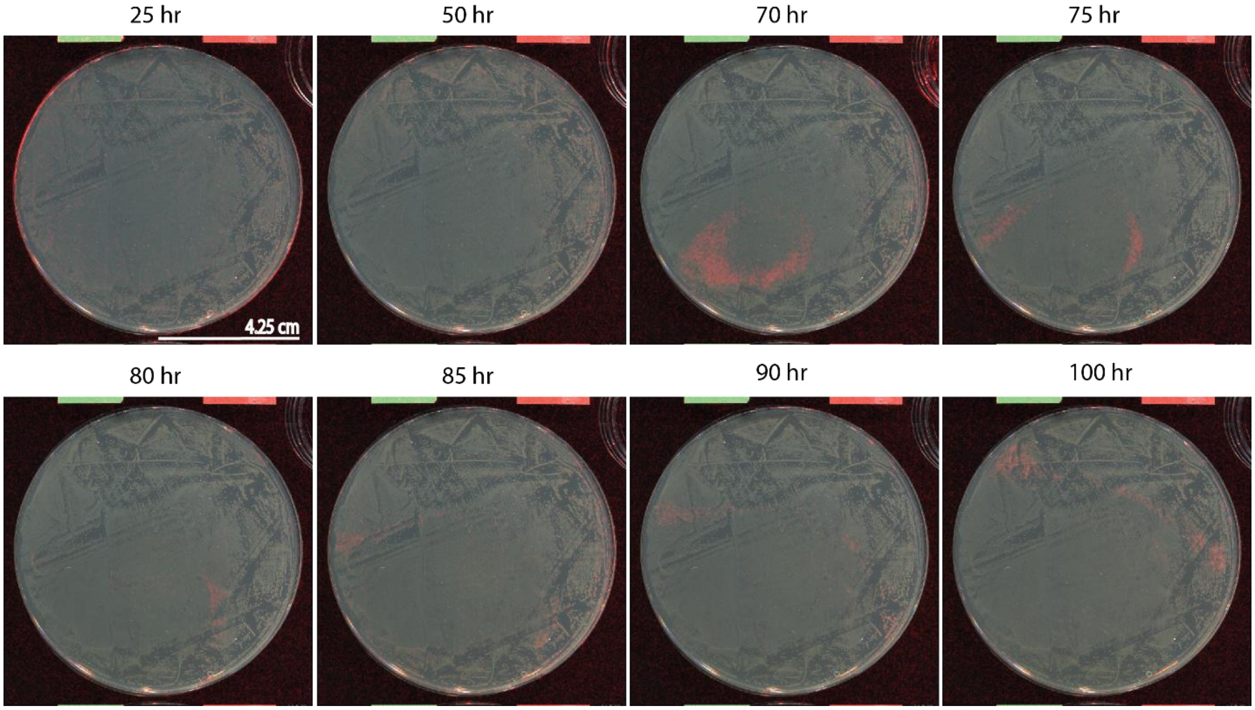

Fig. S3. Detailed snapshots from Video S5 when the prey culture was spread alone on the plate. See Methods for experimental setup (5 biological replicates).

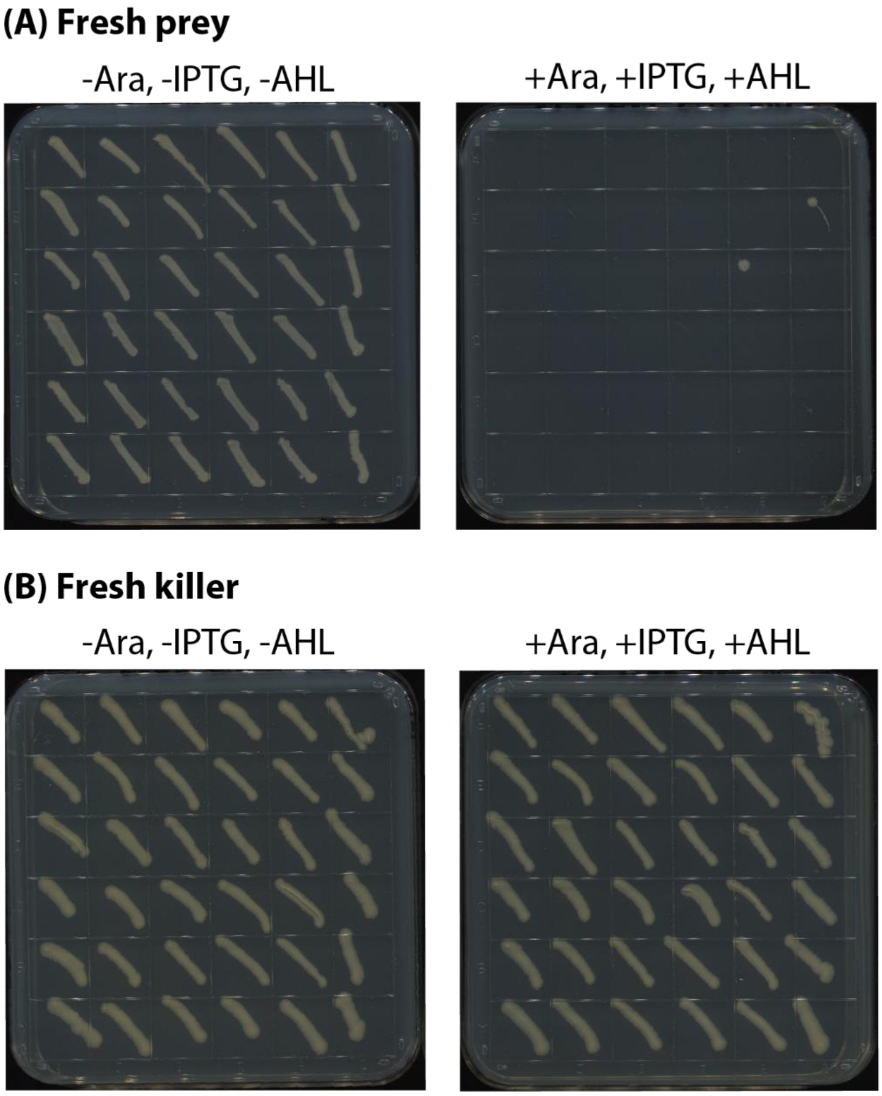

Fig. S4. (A) A representative of the AHL Sensitivity Assay performed on fresh prey cultured overnight, as mentioned in Fig. 2F and Methods. The prey cells grew on the plate without AHL, but failed to grow or showed negligible growth on the plate with AHL. This shows that prey is indeed sensitive to the effects of AHL (n = 36). **(B)** A representative of the AHL Sensitivity Assay performed on fresh killer cultured overnight, as mentioned in Fig. 2F and Methods. The killer cells grew on both the plates with and without AHL, showing that killer is unaffected by AHL (n = 36). All n values correspond to different biological replicates.
